# Oxaliplatin for Metastatic Colon Cancer in a Patient with Renal Failure

**DOI:** 10.4137/cmo.s412

**Published:** 2008-02-09

**Authors:** Kenji Katsumata, Tetsuo Sumi, Tatehiko Wada, Yasuharu Mori, Masayuki Hisada, Hideaki Kawakita, Masanori Enomoto, Shoji Suzuki, Daisuke Matsuda, Akihiko Tsuchida, Tatsuya Aoki

**Affiliations:** Third Department of Surgery, Tokyo Medical University, 6-7-1, Nishi-Shinjuku, Shinjuku, Tokyo, Japan 160-0023

**Keywords:** colorectal cancer, renal failure, oxaliplatin, hemodialysis

## Abstract

**Objective:**

Oxaliplatin, a key part of the standard regimen for colorectal cancer in Western countries, has become available in Japan. In a hemodialysis patient with cecal cancer, we investigated the efficacy, safety, pharmacokinetics, and dialysability of oxaliplatin.

**Methods:**

A 65-year-old man who had cecal cancer was treated with oxaliplatin (40 mg/m^2^) and l-leucovorin(l-LV) (200 mg/m^2^), which were administered simultaneously over 120 min via the side and main arms of a Y-tube, respectively. Then 5-FU (400 mg/m^2^) was administered rapidly via the side tube, followed by 5-FU (2,000 mg/m^2^) over 46 hours via the main tube. The patient had chronic renal failure due to diabetic nephropathy and hemodialysis was performed 3 times a week. Blood samples were collected from the dialyzer before and after each hemodialysis session to examine platinum clearance.

**Results:**

The patient received 3 courses of oxaliplatin before he died of cancer. During hemodialysis, the platinum level fell from 0.32 μg/mL to 0.15 μg/mL.

**Conclusion:**

Since patients with renal failure have various associated disorders and oxaliplatin has a long half-life, it is necessary to obtain more pharmacokinetic data to investigate its accumulation and dialysability during long-term treatment. Such data will assist in treating the rapidly increasing number of hemodialysis patients with colorectal cancer.

## Mini-abstract

The efficacy, safety, pharmacokinetics, and dialysability of oxaliplatin were assessed in a hemodialysis patient with recurrent cecal cancer.

## Introduction

Oxaliplatin (L-OHP) is a key component of the standard regimen for colorectal cancer in Western countries and it has recently become available in Japan. L-OHP has unique characteristics compared with other platinum compounds; it possesses a 1,2-diaminocyclohexane group and a labile oxalate ligand leaving group. This drug has proved effective for colorectal cancer that is unresponsive to other platinum compounds 1.

L-OHP is excreted very slowly in the urine because it shows irreversible protein binding 2. In other countries, this drug has been used to treat patients with renal dysfunction 3,4, but not hemodialysis patients with renal failure. We administered L-OHP to a hemodialysis patient with cecal cancer, and investigated its efficacy, safety, pharmacokinetics, and dialysability.

## Case Report

The patient was a 65-year-old man with a history of diabetes mellitus since the age of 46 years and hypertension since the age of 61. He had been on hemodialysis due to diabetic nephropathy for 4 years.

**Figure 1:**
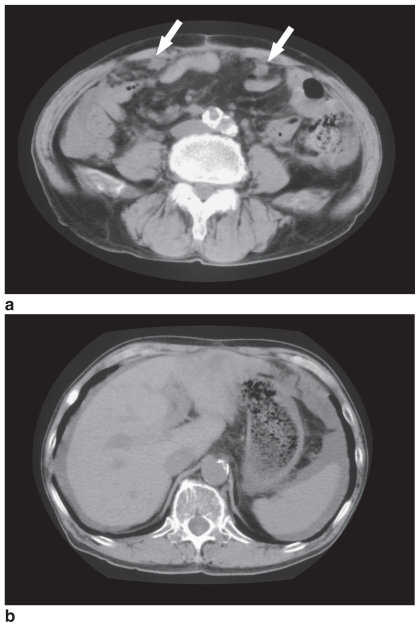
CT scans of a 65-year-old patient with peritoneal dissemination prior to L-OHP administration. (**a**) Nodular masses can be seen in the peritoneal cavity. (**b**) Ascites can be seen around the spleen.

In February 2004, cecal cancer with liver metastases and peritoneal dissemination was diagnosed, and he underwent ileocecal resection plus microwave coagulation therapy for the hepatic metastases. Postoperatively, S-I (a novel oral compound of tegafur, gimestat, and otastat potassium at a ratio of 1:0.4:1 that is aimed at the biochemical modulation of 5-fluorouracil, was administered at a dose of 40 mg. Since peritoneal dissemination was progressive, irinotecan hydrochloride was then administered at a dose of 80 mg every two weeks from February 2005. In May 2005, tumor dissemination showed progression, and tumor marker levels also increased. Abdominal CT scanning revealed ascites and disseminated peritoneal metastases (Fig. 1). Therefore, he was hospitalized for treatment.

On admission, the patient was 165.5 cm tall and weighed 53.0 kg. His conjunctivae were slightly pale. An abdominal tumor was palpable to the right of the umbilicus and intestinal peristalsis was slightly increased.

The laboratory findings on admission were as follows: a white blood cell count of 5.3 × 10^3^/μL, hemoglobin of 8.9 g/dL, platelet count of 18.6 × 10^4^, total protein of 5.8 g/dL, AST of 10 IU/L, ALT of 2 IU/L, T-Bil of 0.38 mg/dL, urea nitrogen of 29.2 mg/dL, creatinine of 6.49 mg/dL, CEA of 27.8 ng/mL, and CA19-9 of 633.0 U/mL.

The patient was treated with reference to the modified FOLFOX6 protocol (mFOLFOX6).10 L-OHP (40 mg/m^2^ for a total dose of 63 mg) and 1-LV (200 mg/m^2^: a total dose of 314 mg) were administered simultaneously over 120 min via the side and the main tubes of a Y-tube, respectively. Then 5-fluorouraci (5-FU) (400 mg/m^2^: a total dose of 628 mg) was administered rapidly via the side tube, followed by 5-FU (2,000 mg/m^2^: a total dose of 3,140 mg) via the main tube over 46 hours. The dose of L-OHP was set at 40 mg/m,^2^ because its area under the concentration vs. time curve (AUC) was reported to increase two-fold when the creatinine clearance was 60 mL/min or less 3. Hemodialysis was performed 3 times a week (Monday, Wednesday, and Friday), and was started 30 min after completing the administration of L-OHP. The free platinum level in plasma ultrafiltrate was measured immediately after the administration of L-OHP, at 15 min afterward, before hemodialysis, immediately after starting hemodialysis, at 30, 60, 90, 120, 150, and 180 min after starting hemodialysis, and after the completion of hemodialysis. To measure the platinum level after administration of L-OHP, blood samples were collected from the dialyzer before and after each hemodialysis session to examine platinum clearance by hemodialysis. Each blood sample was centrifuged immediately after collection, and 1 mL of plasma was subjected to ultrafiltration using an Amicon^®^ filter with a molecular cut-off of 30,000 Da. Then assay of platinum was performed as reported by Gilmour et al. 5.

Hemodialysis was performed for 3 hours at a blood flow rate of 200 mL/min using a TR-2001N dialysis membrane and a BG-1.8U dialyzer (Toray Medical Co., Ltd., Tokyo, Japan). Informed consent was obtained from the patient for the administration of L-OHP and measurement of blood drug levels. Adverse reactions were classified according to Commom Terminology Criteria for Adverse Events version 3.0 15.

To avoid accumulation of L-OHP, it was scheduled to be administered at 3–week intervals. Administration was done on schedule during the second course, but the third course was delayed by 1 week. As adverse events, the neuropathy did not appear in all courses.(Grade 0) and anorexia (Grade 2) and fatigue (Grade 2) developed at 1 week after administration of L-OHP during the first course of therapy. During the second course, anorexia (Grade 3), constipation (Grade 2), and fatigue (Grade 3) occurred, leading to delay of the third course. There was no tumor regression (Fig. 3). On September 26, the patient died due to progression of his cancer.

## Results

**Figure 2:**
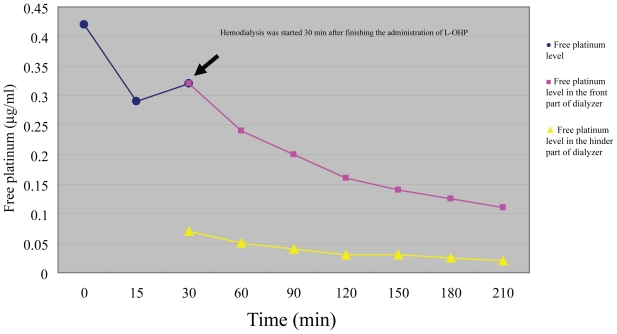
Effect of hemodialysis on platinum concentration in plasma ultrafiltrate.

**Figure 3:**
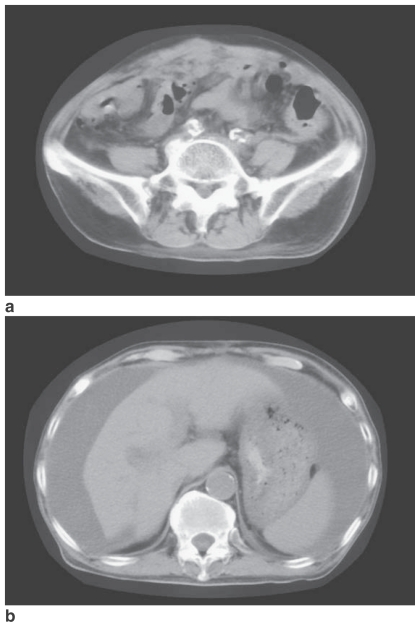
CT scans obtained after L-OHP administration. (**a**) The nodular masses are larger than before. (**b**) Ascites has increased around the spleen.

Figure 2 shows the platinum level in plasma ultra-filtrate after administration of L-OHP. During hemodialysis, the platinum level decreased from 0.32 μg/mL at baseline to 0.24, 0.2, 0.16, and 0.14 μg/mL, showing a decline of about 50%. The removal rate of platinum by the dialyzer was 80% irrespective of the time after starting hemodialysis.

## Discussion

The number of patients on hemodialysis due to renal failure has been increasing steadily in Japan. In 2000, there were 206,134 such patients, and 18,938 of them died of malignant diseases. Since malignancy ranks fourth among the causes of death for hemodialysis patients, treatment of cancer in these patients is a major problem 6.

At present, there are no established chemotherapy for the treatment of malignant diseases having hemodialysis. In 1998, the total number of cancer deaths was 284,000, and colorectal cancer accounted for 12.1%, ranking third after lung cancer and gastric cancer 7–10.

For these reasons, effective chemotherapy for colorectal cancer in hemodialysis patients is needed.

In Western countries, combination chemotherapy with 5-FU, 1-LV, and L-OHP (FOLFOX) is commonly used as first-line treatment for colorectal cancer 11. L-OHP has also been approved in Japan recently, so that 5-FU, 1-LV, L-OHP, and irinotecan (used in Western countries as standard chemotherapy for colorectal cancer) can now be used in Japan.

We administered reduced-dose mFOLFOX6 to our hemodialysis patient with cecal cancer that was refractory to S-1 and irinotecan, and measured the platinum level over time. L-OHP is a third-generation platinum derivative that is eliminated via the kidneys. According to pharmacokinetic studies, the protein-binding rate of L-OHP is 85%–88%, which is similar to that of other platinum compounds. However, L-OHP shows irreversible binding to erythrocytes and thus is slowly eliminated from the body, with the t_1/2_ for elimination from erythrocytes being 400–600 hours ^2^. Also, the plasma t_1/2_ for platinum was reported to be as long as 392 hours in men without liver metastasis who were under 65 years old. These findings suggest that, unlike other platinum preparations, Reanal function has little influence on L-OHP toxicities and the long terminal half lives are likely due to inactive species of L-OHP as exatapolated from the absence of increased toxicity in spite of such ultrafilterable platinum retention. Comparison between patients classified into groups on the basis of a creatinine clearance (Ccr) of 60 mL/min, has revealed significant differences in the total dose of L-OHP and the t_1/2_ (β), while there was no difference of adverse events 4. A group of investigators at the NCI (U.S.A) also administered this drug to patients with a Ccr of 20 mL/min. They found no influence on the development of adverse events, although the AUC was elevated, but the number of subjects was small 12.

It has been reported that the standard dose of 5-FU can be administered to hemodialysis patients, because 80% or more of this drug is metabolized and inactivated in the liver, most of the metabolites are eliminated in the expired air, the protein-binding rate is low, and it is dialyzable 13. However, it was also reported that blood drug levels were higher than those observed in healthy volunteers when 5-FU was administered orally to patients with renal failure or was given by rapid intravenous injection 14.

Although 5-FU levels were not examined in our patient, the platinum level (released from L-OHP) was 0.42, 0.29, and 0.32 μg/mL immediately after administration, at 15 min after the administration, and just before hemodialysis, respectively. The platinum level before the dialyzer circuit was 0.11–0.32 μg/mL. A pharmacokinetic study of L-OHP performed in Japanese subjects showed that the blood level of platinum around 0.2 μg/mL for 24 hours from 3 hours after administration. These results suggest that the blood platinum level may have a similar profile to that in patients who are not on dialysis, although no definite conclusions can be drawn because we have only examined pharmacokinetic parameters in one patient. Although the dose of L-OHP was reduced to 40 mg/m^2^, the AUC was increased in agreement with previous reports on patients who had impaired renal function. The platinum level after the dialyzer circuit was 0.02–0.07 μg/mL, which suggests the possibility of L-OHP being dialysable. Considering that the t_1/2_ γ of L-OHP was 392 hours, that the 24-hour urinary excretion rate after its administration at 130 mg/m^2^ was 33.9 ± 8.8% of the total platinum dose in the Japanese phase I study, and that the 120-hour urinary excretion rate was reported to be 53.8 ± 9.1% overseas, this drug is considered to be tolerable for patients on hemodialysis 3 times a week.

It was difficult to evaluate adverse events because our patient had advanced cancer. However, gastrointestinal symptoms appeared to be slight, but persistent. Although their relationship to renal failure could not be properly evaluated, considering the possible accumulation of L-OHP, it may be more appropriate to administer this drug at intervals of at least 3 weeks. Since patients with renal failure have various associated disorders and the blood half-life of L-OHP is long, it will be necessary to collect more pharmacokinetic data to investigate its accumulation and dialysability in patients receiving this drug over the long term. Such data will assist in treating the rapidly increasing number of hemodialysis patients with colorectal cancer.
